# How Experts’ Use of Medical Technical Jargon in Different Types of Online Health Forums Affects Perceived Information Credibility: Randomized Experiment With Laypersons

**DOI:** 10.2196/jmir.8346

**Published:** 2018-01-23

**Authors:** Maria Zimmermann, Regina Jucks

**Affiliations:** ^1^ Institute for Psychology in Education Department of Psychology and Sport Science University of Münster Münster Germany

**Keywords:** trust, health communication, social media, information seeking behavior, language

## Abstract

**Background:**

Online health forums are widely used, but the quality of advice differs as much as the knowledge backgrounds of the audience members who receive the advice. It is important to understand how people judge the information given online. In line with the communication accommodation theory (CAT), online forums represent specific social contexts of communication which can present either accommodative or nonaccommodative language to an audience. Accordingly, use of accommodative or nonaccommodative language might affect people’s perceived trust in the communicator.

**Objective:**

The objective of this study was to investigate how experts who use accommodative (vs nonaccommodative) language are evaluated by passive users of an online forum.

**Methods:**

Participants (n=98) took part in an online experiment and read experts’ posts about 10 nutrition myths. Following a 2 x 2 mixed design, experts’ posts were written using either low or high amounts of medical technical jargon (MTJ) (within factor) and were directed at different audiences (mainly other medical experts [in a professional forum] vs a user group mainly comprising laypersons [in an advisory forum]) (between factor). Accommodation occurred where experts used high amounts of MTJ to address other medical experts in the professional forum; it also occurred when experts used low amounts of MTJ to address laypersons in the advisory forum. Conversely, nonaccommodation occurred when experts used high amounts of MTJ in the advisory forum and low amounts of MTJ in the professional forum. In each condition, participants evaluated the credibility of the information, the trustworthiness of the experts, and the accommodation by the experts.

**Results:**

Overall, participants judged the credibility of information to be higher when experts used MTJ that was accommodative to the designated audience, *F*_1,95_=3.10, *P*=.04, η_p_^2^=.031. In addition, participants judged the experts in professional forums to be more trustworthy than experts in advisory forums (all *F*_1,96_≥3.54, *P* ≤.03, η_p_^2^≥.036). Moreover, participants rated experts who used high amounts of MTJ to have higher competence (*F*_1,96_=37.54, *P*<.001, η_p_^2^=.28], lower integrity (*F*_1,96_=10.77, *P*=.001, η_p_^2^=.101), and lower benevolence (*F*_1,96_=9.75, *P*=.002, η_p_^2^=.092), as well as to have lower perceived accommodation to the audience (all *F*_1,96_≥72.17, *P*<.001, η_p_^2^≥.43) compared with experts who used low MTJ.

**Conclusions:**

To provide health information online that is perceived as credible, experts should consider using similar language as the language used by the addressed audience. As it is often impossible to determine the exact makeup of an online audience, further research might investigate whether having experts explicitly declare which audience they intend to address can help people to more reliably assess an expert’s trustworthiness. Furthermore, as people assess information differently depending on the context of online communication, it would be valuable for research to consider other aspects of the context beyond those of the audience.

## Introduction

### Trust in Online Health Forums

For a member of the general public trying to find specific advice in an online forum, determining who to believe and on which information to rely is fraught with a number of challenges [1,2]. The main challenges are related to, on the one hand, the quality of information and the lack of quality control [3,4], and, on the other hand, the difficulty that laypersons have in adequately assessing, understanding, and judging the given information [2,5-7]. As people are increasingly using the Internet to obtain nutrition information [8,9], it is becoming more important to understand the associated challenges people face in accurately judging the information and the information providers.

To get correct and understandable health information, laypersons need to rely on qualified information providers, as their own expertise might lack in specific topics [10]. By relying on the information from an information provider, the layperson risks losing something or being disadvantaged in some way if the information is false [11-13]. Although people differ in their willingness to rely on others as a source of information and to judge the persuasive quality of a source (ie, epistemic trustworthiness) [14], how people determine if something is credible relates more to the objective features of the information itself [15]. However, judging credibility also involves subjective judgments of, for instance, a source’s trustworthiness, expertise, and attractiveness [5,6]. Therefore, in this paper, trustworthiness and credibility are used as concepts dependent on each other, whereas epistemic trustworthiness focuses on the reliability of experts and credibility focuses on the reliability of the provided information.

Although finding health information online still poses risks to information seekers of being misinformed by unreliable information or information providers, having access to online information can be empowering. People want to find understandable health information online [7,16]. Moreover, if people can find understandable information and comprehend content-related issues, they may make better health-related decisions [2,17]. Accordingly, the most common reason people visit online health forums (eg, about nutrition) is to search for information [18,19].

Online health forums are visited by users from wide-ranging communities with various knowledge backgrounds (ie, medical professionals and medical novices, as well as patients and members of the general public). These forums allow users to exchange information, regardless of a user’s knowledge of or expertise in a certain subject [18,20,21]. Thereby, the extent of professionals’ engagement in forum discussions differs between forums [22], and the main user group is likely to be different in different forums. In some forums, professionals *discuss among themselves* [23,24], whereas in others, professionals advise a user group made up mainly of laypersons [25,26], and in others still, laypersons primarily exchange information with other laypersons [27-29].

However, in any online forum, the actual audience extends beyond the forum’s designated (intended) audience; many users *lurk*, that is, they visit forums only passively to receive information rather than to post their own contribution [20,30]. Accordingly, several forms of expert-layperson communication may occur, such that different numbers of people with different levels of expertise are involved (eg, one expert is communicating with one layperson while simultaneously one expert is communicating with many others). Moreover, in online forums, these types of expert-layperson communications differ from offline expert-layperson communication. First, the health information provided in online forums reaches a broad layperson public [20], because online forums are not closed and hence can be accessed by a large public. Thus, even when an expert is communicating directly with a specific person in a forum, many other visitors to the forum (users) and, potentially, even the entire public could read the provided information. Second, the way in which experts use language becomes even more important in online forums because most often their written language is the only obvious cue that can be used by forum visitors to assess the expert’s trustworthiness [31,32]. Regarding the used language in expert-layperson communication, it is particularly challenging when experts use technical jargon [33]. Using too much technical jargon could prevent the main goal to impart knowledge, as people with less background knowledge might not understand technical jargon [33,34] or find it less credible [35].

Although in online forums the forms of terminology vary from everyday language to highly technical jargon, users may expect a certain type of wording depending on the designated audience of forum types. Accordingly, users would need to recognize not only the specific language use of experts but also the forum context. Therefore, the study reported here addresses passive forum users’ judgment of experts’ trustworthiness and credibility based on the way the experts used language in different forum contexts. Online information seekers are at risk to interpret even accurate health information incorrectly (leading to misconceptions, misinformation, and harmful decisions) if they do not recognize the context cues that indicate a certain piece of health-related information as intended for specific audiences (also described as *context deficit* [2,4]). Similarly, the communication context plays a crucial role in the communication accommodation theory (CAT) [36], and it not only influences the communication itself (eg, the language style used) but also affects the perception of communication as well as the communication outcome.

### Communication Accommodation Theory Online

Following the CAT [36,37], online health communication can be considered as an interplay between (1) the people communicating, (2) the language they are using, and (3) the context (ie, the immediate situation where communication takes place). For instance, the specific audience and the interlocutors’ languages determine what variants of language are used [38]. People adjust their language using 2 key strategies: accommodation and nonaccommodation.

Accommodation relates to the process of using similar language relative to the perceived language of the audience, and it can be regarded as a tactic for conforming to the audience and emphasizing social belongingness [39]. Moreover, accommodation can lead to higher comprehension by the audience, as adapting one’s language to that of the audience can occur after one perceives the audience’s background knowledge [10,40]. Similarly, when an expert uses high amounts of medical technical jargon (MTJ) in a professional forum, as well as when they use low amounts of MTJ in an advisory forum, this is considered to be accommodation, as it refers to using similar language relative to the perceived language and background knowledge of the audience.

On the other hand, nonaccommodation relates to the process of using different language relative to the perceived language of the audience. In this context, nonaccommodation can be regarded as a tactic for showing that one has distinct values from the interlocutor [41]. Importantly though, nonaccommodative use of MTJ could inhibit comprehension and knowledge transfer [34]. Accordingly, when an expert uses high amounts of MTJ in an advisory forum or low amounts of MTJ in a professional forum, this can be considered nonaccommodation, as it refers to using different language relative to the perceived language of the audience.

Research on health communication often focuses on 2 essential communication styles used by doctors (ie, doctor-centered vs patient-centered communication) and their impact on patients’ evaluation of doctors’ and patients’ behavioral changes [42,43]. Although doctor-centered communication is characterized by rational-cognitive proceedings without paying much attention to a patient’s needs and feelings, patient-centered communication is more open, non-directive, and aims to actively engage patients by focusing on their psychological and social situation [43]. In patient-centered communication, doctors would tend to avoid using medical terms, as this takes patients’ needs for understanding information and satisfaction with doctor-patient communication into account [44,45]. However, doctors often use more medical terms compared with even well-educated patients [46].

According to previous research on the effectiveness of communication styles, information providers that used a more doctor-centered communication style were perceived by participants to be less competent, less empathetic, and less trustworthy, and they led participants to report fewer changes in attitude compared with when information providers used a more patient-centered communication approach [43,47,48]. However, this does not mean that all patients equally prefer a patient-centered communication. Instead, a study on patients’ preferred communication style showed that there are also 30.8% (77/250) patients who preferred a more doctor-centered communication style. Moreover, the remaining patients who preferred a more patient-centered communication style characterized their own doctor as patient-centered, appreciated when the doctor showed interest in the patient as a person, and were younger than 65 years [49]. Thus, doctors’ communication styles should try to match patients’ communication preferences to enhance patients’ satisfaction and to promote patients’ health care [48].

Accordingly, a match between a doctor’s and a patient’s communication style can be considered accommodation, and a mismatch can be considered nonaccommodation. For instance, when focusing on the wording, medical students answering an email inquiry were influenced by the technicality of inquiries and adapted their use of words to the level of technicality in the inquiry [50,51]. Similarly, users in online health forums adapted their language of replies to the language of the inquiry [52], and medical professionals of 7 major German health portals used no medical terms in reply to a question only when that question itself did not contain any medical terms [53]. In summary, these semiprofessionals and professionals used similar language styles, relative to those used in the health inquiries (ie, accommodation). In contrast, medical students in early semesters with high biomedical orientation (ie, having concepts of scientific and evidence-based medicine) replied more scientifically and less emotionally to all wordings of patients’ queries and, hence, did not use wording similar to what the patients used (ie, nonaccommodation) [39].

### This Study and Hypothesis

This study sheds light on whether passive forum users are sensitive to experts’ use of accommodative or nonaccommodative language; we assessed this by varying the designated audience of an online forum and by varying the amount of MTJ (ie, the wording of information) experts used in their responses to forum inquiries. Specifically, we wanted to know how these 2 factors influenced users’ judgments of the experts’ trustworthiness and credibility. In face-to-face settings, people seem to think it is more appropriate for a speaker to accommodate their language to match that of the addressed audience compared with when the speaker does not accommodate [54]. In addition, accommodative language use is also strongly accompanied with people’s perceived credibility [55]. Is this also true for information processing in online health forums?

Research on health communication suggests that professionals should consider information seekers’ needs to promote information seekers’ understanding and health [42,43,47-49]. Accordingly, professionals’ communication styles can match the information seekers’ communication styles and, therefore, reflect these needs [50-53]. Hence, the accommodative use of MTJ should increase each passive user’s confidence that audiences are receiving appropriate information from competent advice givers. Thus, we hypothesized that accommodative language use would lead to higher credibility and trustworthiness ratings than nonaccommodative language use (ie, the interaction effect of the designated audience in forum types and amount of MTJ). We assumed that in a professional forum, a high amount of MTJ would lead to higher ratings for the dependent variables of credibility of information [56], trustworthiness of experts [14], and accommodation by experts [33] compared with when a high amount of MTJ was used in an advisory forum. Similarly, we assumed that in an advisory forum, a low amount of MTJ would lead to higher ratings for the dependent variables compared with when a low amount of MTJ was used in a professional forum.

As the aim of the investigation was to examine whether people judge the credibility of information and an expert’s trustworthiness according to the expert’s accommodative (or nonaccommodative) use of MTJ in 2 different types of forums, we had participants assess not only the expert’s trustworthiness and credibility of information but also the perceived accommodation by the experts to the audience.

## Methods

### Design

We applied a 2 × 2 mixed design, with the independent factors being designated audience of the forum type (in a *professional forum*, the main audience is other medical experts who are exchanging information with each other, and in an *advisory forum*, the main audience is laypersons) and the amount of MTJ used (high vs low).

The designated audience was manipulated between conditions. Professional forums were introduced as forums that are mainly used by medical experts to exchange technical content and to take part in scientific discussions. In contrast, advisory forums were introduced as forums that are mainly used by patients and other nonprofessionals to inform themselves about health-related questions, to take part in explanation-oriented discussions, and to receive answers from medical professional (see Multimedia Appendix 1). Both introductions for forum types declared that the following posts were written by experts. Hence, the information providers in both forums had the same status as experts, as people judge trustworthiness and the credibility of information based on the information providers’ expertise [57]. In addition to describing the forum type in the overall introduction, every post about a nutrition myth was introduced by a slightly different short description about the forum type (according to the condition) to promote participants’ awareness of the forum context (Multimedia Appendix 2).

MTJ was varied within subjects; either the expert’s post included language using Greek or Latin expressions or it included synonymous everyday German expressions (see Multimedia Appendix 3). In addition, 1 complex sentence from the version containing the high level of MTJ was split into 2 less complex sentences for the version having a low level of MTJ.

### Procedure

Participants completed an online survey via the platform Questback EFS Survey and answered questions regarding demographic and control variables before they were randomly assigned to one of the forum type conditions (professional forum vs advisory forum). Depending on which condition they were assigned to, they either read an introduction explaining that the following posts came from a forum used mainly by medical professionals to discuss among themselves or they read an introduction explaining that the posts came from a forum in which medical professionals advice a user group mainly made up of laypersons. This overall introduction of forum type was presented for at least 16 seconds to ensure participants saw it. Every participant had to complete 10 survey sites, each of which included a short description of the specific forum type, a moderated question to introduce the nutrition topic, and a screenshot of the expert’s post. Posts were presented in a fixed order to control the influence of different nutrition myths.

### Materials

Nutrition myths were chosen as the content domain for this study because it is an interesting (hot) topic [58,59]. All nutrition myths (eg, coffee and dementia, olive oil and cardiac infarction, healthy number of eggs; 10 topics in total, see Multimedia Appendix 3) were based on our online searches for frequent and typical nutrition myths in online forums. To explain the science behind the myths, we used the textbook Nutrition Science by de Groot and Farhadi [60], and we formulated certain scientific limitations by using relativizations (eg, there is some evidence). To make these texts look like real experts’ posts, one online forum was created; texts were posted; and the date, amount, and origin of posts were made unrecognizable (screenshot of a survey site showing an expert’s post, see Multimedia Appendix 4). Overall, each participant received 10 experts’ posts about a nutrition myth (5 high in amount of MTJ and 5 low in amount of MTJ). Posts did not differ in average length. In the German language, both forms of medical terminology exist: everyday medical terms in German as well as medical technical terms that stem from Greek or Latin (eg, blood pressure-lowering vs antihypertensive) [10,61]. MTJ not only contains medical technical terms that stem from Greek or Latin but also contains highly complex sentences [62]. Hence, the experts’ posts at the high MTJ level included medical technical terms (Greek and Latin) and 1 complex sentence, whereas the experts’ posts at the low MTJ level contained no medical technical terms (they were replaced with everyday medical synonyms) and no complex sentences. Synonymous usage of these terms was ensured by referring to a medical dictionary [63] (notice that in German, nearly every everyday medical term has a Greek or Latin synonym [64]).

### Participants

An analysis of power (assuming 1 − ß=.80; Cohen f=.25) was used in advance to determine the sample size (n=92). In total, data from 106 participants were collected. Three participants were excluded from data analysis because at the end of the survey they specified that they did not want to provide their data for research purposes. Three additional participants were excluded because their time to complete the survey (ranging from 267 min to 870 min) took more than one standard deviation (SD) above the overall mean duration of all participants (mean 49.3 min, SD 90.6); we decided to exclude these participants because they were likely engaged in other activities when they were answering the survey. Furthermore, 2 more participants were excluded because they reported professional prior knowledge—1 studied health promotion and 1 was a trained paramedic.

Hence, in sum, data from 98 participants (72 females) aged 18 to 54 years (mean 25.18, SD 6.33) were analyzed. A total of 92 participants indicated German as their first language (the other 6 participants had been speaking German for an average of 11.17 years [SD 5.31]). Moreover, 93 participants specified that they were currently studying or had studied at the university level and hence had a university-entrance diploma. On average, they reported studying for 4.77 semesters (SD 3.04). Furthermore, 52 majored in psychology, and the others came from various disciplines (19 from law, economics, and social science; 8 from natural science, engineering science, and mathematics; 8 from linguistic and cultural studies; 5 from teacher training; and 1 from sports science).

Participants reported that they used a computer for an average of 29.06 (SD 20.02) hours per week and the Internet for an average of 26.71 (SD 22.75) hours per week. A total of 81 out of 98 participants (83%) reported that they used text messaging (short message service, SMS) daily. Regarding email use, 39 out of 98 participants (40%) reported using it daily; 49 participants (50%) at least several times per week. In addition, most participants did not regularly visit forums: 72 out of 98 participants (74%) stated they used a forum less than once per month. Furthermore, participants reported that their prior knowledge of and motivation to be informed about the topic *health and nutrition* (from 1=I agree to 5=I disagree) to be on average 2.33 (SD 0.77); the 4 items they scored themselves on were as follows: I deal a lot with the topic *health and nutrition*; To me, it is important to eat healthy; I read a lot about *health and nutrition* on the Internet; and I am familiar with the topic *health and nutrition*.

Participants were invited by a link on several online platforms run by a big German university and a German remote university. They were automatically excluded from the survey if they had stated that they were currently studying or had studied medicine or nutrition science. We excluded them because these students’ high amount of prior knowledge may have influenced their judgments of credibility and trust in the information within the forum posts. A total of 47 participants were assigned to the professional forum condition and 51 were assigned to the advisory forum condition; they received a 10€ voucher as reimbursement. In addition, the survey access was automatically denied when participants attempted to use a mobile phone; thus, it allowed us to control the screen size of the devices used. The average duration of completed surveys was 37.99 min (SD 15.70).

### Dependent Measures

This study employed 3 dependent variables: credibility of information, trustworthiness of experts, and perceived accommodation by experts.

#### Credibility of Information

Participants were asked to indicate whether they agreed with the given information (How much would you agree with the answer?) and to judge the credibility of the information using 4 additional items adopted from a measurement scale assessing trust in journalism [56]. We further added the item *I would like to ask someone else*. Overall, these 6 items yielded an internal consistency of Cronbach alpha=.86. Participants rated items on a 5-point Likert scale from 1 (I strongly disagree) to 5 (I strongly agree).

#### Trustworthiness of Experts

Trustworthiness of experts was assessed by Muenster Epistemic Trustworthiness Inventory (METI) [14]. METI was designed to measure the epistemic trustworthiness of unknown sources and, therefore, is useful in online settings, because online information about the source is often scarce. METI consists of 14 items and is composed of 3 subscales. The subscale Expertise reflects the participant’s perception of the expert as truly knowledgeable, intelligent, and highly trained in her domain. The subscale Integrity is related to the expert’s good character and values, and reflects the participant’s perception of the expert as a person who is acting in line with norms. The Benevolence subscale represents the participant’s perception of whether the expert acts in accordance with the interest of others.

To rate the trustworthiness of each expert, participants used 7-point scales (eg, 1=competent to 7=incompetent) containing acronym adjectives that represented the subscale to which the items belonged. The item unselfish-selfish (not included in the revised version of METI) was added. Internal consistencies were Cronbach alpha=.95 for the subscale Expertise (6 items), Cronbach alpha=.89 for Integrity (5 items), and Cronbach alpha=.87 for Benevolence (4 items). Overall, all 15 items yield a Cronbach alpha=.95 (METI Score).

#### Perceived Accommodation by Experts

The perceived accommodation by experts was assessed using an adaption of the recipient orientation scale (ROS) [33], which refers to how experts are perceived to adjust their language to an audience. The dimension Audience Design reflects the perceived willingness of the expert to adapt to the audience (eg, the expert can imagine how it is to know little about this topic). The dimension Subjective Comprehension is related to the participant’s self-reported understanding about the topic at hand (eg, I understood the content). In addition, the dimension Emotional Evaluation is related to the participant’s self-reported liking of reading the expert’s posts (eg, it is exciting to read the response of the expert). Overall, all 13 items yielded a Cronbach alpha=.90. Internal consistencies were Cronbach alpha=.81 for Audience Design (6 Items), Cronbach alpha=.72 for Subjective Comprehension (3 Items), and Cronbach alpha=.73 for Emotional Evaluation (4 Items).

## Results

### Preliminary Analysis

In a preliminary analysis, we assessed the differences between subsamples of each forum condition for the expected control variables. A multivariate analysis revealed no differences in groups regarding any of the participant demographic variables, their reported usage of forums, the Internet or computers, or the 4 items that assessed self-reported general prior knowledge, all *F*_1,91_<3.78, *P*>.06. Moreover, a second preliminary multivariate analysis included the within factor MTJ and revealed no differences between the forum conditions regarding the 3 control items that assessed subjective familiarity (I’m familiar with the content of the response), subjective complexity (This is a complex issue), and interest (The response is interesting) for each topic. Hence, none of these items were included as a control variable in the following analysis.

An alpha level of .05 was set for a multivariate variance analysis with repeated measures, where MTJ was the within subject factor and designated audience was the between subject factor. All tests were one sided. For each participant, we used averaged values of the dependent variables for all the 5 high MTJ and all the 5 low MTJ responses; hence, for each participant, 2 average values—one for the low MTJ condition and one for the high MTJ condition—were used for further analysis.

### Credibility of Information

**Figure 1:**
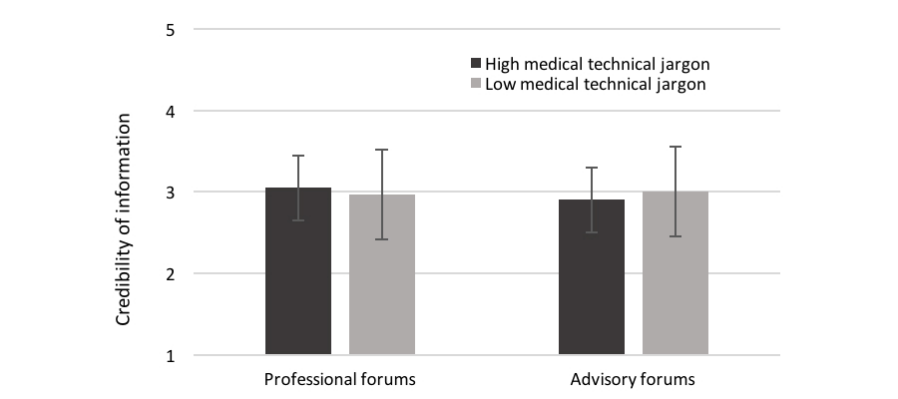
Interaction of medical technical jargon and designated audience in terms of credibility of information (1=strongly disagree to 5=strongly agree).

There were no main effects of MTJ (*F*_1,96_=0.07, *P*=.40) and of designated audience (*F*_1,96_=0.52, *P*=.24) regarding credibility. However, the multivariate analysis yielded a significant interaction of MTJ and designated audience (*F*_1,95_=3.10, *P*=.04, η_p_^2^=.031), with higher credibility judgments given for the condition that used high amounts of MTJ in professional forums (mean 3.05, SD 0.38) compared with the condition of using low amounts of MTJ in professional forums (mean 2.97, SD 0.47). Conversely, in advisory forums, low amounts of MTJ (mean 3.00, SD 0.60) led to higher credibility judgments than high amounts of MTJ (mean 2.90, SD 0.44). Figure 1 illustrates the interaction of MTJ and designated audience in terms of credibility of information.

### Trustworthiness of Experts

Both main effects showed significance: MTJ affected all the 3 subscales of the METI; Expertise *F*_1,96_=37.54, *P*<.001, η_p_^2^=.28, Integrity, *F*_1,96_=10.77, *P*=.001, η_p_^2^=.101, and Benevolence, *F*_1,96_=9.75, *P*=.002, η_p_^2^=.092. High amounts of MTJ led to higher expertise ratings (mean 2.82, SD 0.80) than did low amounts of MTJ (mean 3.19, SD 0.79). However, low amounts of MTJ led to higher Integrity (mean 3.42, SD 0.62) and Benevolence ratings (mean 3.47, SD 0.67) compared with conditions of high amounts of MTJ (Integrity: mean 3.57, SD 0.55; Benevolence: mean 3.66, SD 0.57).

The designated audience affected the METI score *F*_1,96_=3.54, *P*=.03, η_p_^2^=.036, and the subscales of Integrity, *F*_1,96_=3.54, *P*=.03, η_p_^2^=.036, and Benevolence, *F*_1,96_=6.11, *P*=.001, η_p_^2^=.06. Thereby, experts in professional forums were judged as being more trustworthy (mean 3.21, SD 0.63), of greater integrity (mean 3.39, SD 0.63), and more benevolent (mean 3.43, SD 0.65) than experts in advisory forums (METI score: mean 3.40, SD 0.57; Integrity: mean 3.59, SD 0.53; Benevolence: mean 3.70, SD 0.56). There was no significant interaction of MTJ and designated audience regarding trustworthiness, all *F*_1,95_<1.84, *P*>.09.

### Perceived Accommodation by Experts

Significant main effects of MTJ and designated audience were found for perceived accommodation by experts. MTJ affected the overall score of the ROS and all subscales, all *F*_1,96_≥72.17, *P*<.001, η_p_^2^≥.43, where more expert accommodation was found in the low MTJ condition. The analysis also revealed a significant main effect of designated audience on the subscale Emotional Evaluation, *F*_1,96_=3.35, *P*=.04, η_p_^2^=.03, where participants ascribed emotional evaluation to experts in professional forums (mean 3.10, SD 0.51) than to experts in advisory forums (mean 2.92, SD 0.58). There were no significant interaction effects of MTJ and designated audience regarding perceived accommodation by experts: all *F*_1,95_<2.61, *P*>.06.

Table 1 shows descriptive values for the factors MTJ and designated audience for each of the dependent variables. Table 2 shows values for the multivariate analysis of variance, including the within factor of MTJ and the between factor of designated audience for each of the dependent variables.

**Table 1:** Descriptive statistics.

Factors			Professional forum, mean (SD)	Advisory forum, mean (SD)	Total, mean (SD)
**Trustworthiness of experts**					
	**METI**^a^ **score**				
		High MTJ^b^	3.16 (0.63)	3.42 (0.50)	3.30 (0.58)
		Low MTJ	3.26 (0.63)	3.41 (0.64)	3.34 (0.63)
		Total	3.21 (0.63)	3.42 (0.57)	3.31 (0.60)
	**Expertise**				
		High MTJ	2.71 (0.81)	2.92 (0.78)	2.82 (0.80)
		Low MTJ	3.12 (0.79)	3.26 (0.79)	3.19 (0.79)
		Total	2.91 (0.80)	3.11 (0.79)	3.01 (0.79)
	**Integrity**				
		High MTJ	3.44 (0.62)	3.70 (0.46)	3.58 (0.55)
		Low MTJ	3.34 (0.63)	3.49 (0.60)	3.42 (0.62)
		Total	3.39 (0.63)	3.59 (0.53)	3.49 (0.58)
	**Benevolence**				
		High MTJ	3.48 (0.63)	3.83 (0.45)	3.66 (0.57)
		Low MTJ	3.38 (0.68)	3.56 (0.67)	3.47 (0.67)
		Total	3.43 (0.65)	3.70 (0.56)	3.56 (0.61)
**Credibility of information**					
		High MTJ	3.05 (0.38)	2.90 (0.44)	2.97 (0.42)
		Low MTJ	2.97 (0.47)	3.00 (0.60)	2.99 (0.54)
		Total	3.01 (0.42)	2.95 (0.52)	2.98 (0.47)
**Perceived accommodation by experts**					
	**ROS**^c^ **score**				
		High MTJ	2.79 (0.42)	2.62 (0.49)	2.70 (0.46)
		Low MTJ	3.51 (0.46)	3.48 (0.44)	3.49 (0.45)
		Total	3.15 (0.44)	3.05 (0.47)	3.10 (0.45)
	**Audience design**				
		High MTJ	2.58 (0.46)	2.43 (0.53)	2.51 (0.49)
		Low MTJ	3.43 (0.45)	3.49 (0.42)	3.46 (0.43)
		Total	3.01 (0.45)	2.96 (0.48)	2.98 (0.47)
	**Subjective comprehension**				
		High MTJ	3.25 (0.64)	3.10 (0.64)	3.17 (0.64)
		Low MTJ	3.80 (0.55)	3.70 (0.55)	3.75 (0.55)
		Total	3.52 (0.60)	3.40 (0.60)	5.16 (0.60)
	**Emotional evaluation**				
		High MTJ	2.76 (0.49)	2.54 (0.57)	2.64 (0.52)
		Low MTJ	3.41 (0.55)	3.30 (0.60)	3.35 (0.58)
		Total	3.10 (0.52)	2.92 (0.58)	6.00 (0.55)

^a^METI: Muenster Epistemic Trustworthiness Inventory.

^b^MTJ: medical technical jargon.

^c^ROS: recipient orientation scale.

**Table 2:** Multivariate analysis of variance including the factors medical technical jargon (within subjects) and designated audience (between subjects) for each of the dependent variables.

Factors and tests^a^			Degrees of freedom	*F*	*P*^a^	η_p_^2^
**MTJ**^b^ **(within)**							
	**Trustworthiness of experts**					
		METI^c^ score	1	0.951	.17	0.010
		Expertise	1	37.543	<.001	0.281
		Integrity	1	10.772	<.001	0.101
		Benevolence	1	9.751	.001	0.092
	Credibility		1	0.068	.40	0.001
	**Perceived accommodation by experts**					
		ROS^d^ score	1	178.611	<.001	0.650
		Audience design	1	219.130	<.001	0.695
		Subjective comprehension	1	72.172	<.001	0.429
		Emotional evaluation	1	11.492	<.001	0.537
**Forum type (between)**							
	**Trustworthiness of experts**					
		METI score	1	3.537	.03	0.036
		Expertise	1	1.469	.11	0.015
		Integrity	1	3.537	.03	0.036
		Benevolence	1	6.113	.01	0.060
	Credibility		1	0.523	.24	0.005
	**Perceived accommodation by experts**					
		ROS score	1	2.047	.08	0.021
		Audience design	1	0.486	.24	0.005
		Subjective comprehension	1	1.541	.11	0.016
		Emotional evaluation	1	3.346	.04	0.034
**MTJ forum type**							
	**Trustworthiness of experts**					
		METI score	1	1.425	.12	0.015
		Expertise	1	0.405	.26	0.004
		Integrity	1	1.575	.11	0.016
		Benevolence	1	1.841	.09	0.019
	Credibility		1	3.101	.04	0.031
	**Perceived accommodation by experts**					
		ROS score	1	1.497	.11	0.015
		Audience design	1	2.609	.06	0.026
		Subjective comprehension	1	0.178	.34	0.002
		Emotional evaluation	1	0.741	.20	0.008

^a^All tests were one-sided.

^b^MTJ: medical technical jargon.

^c^METI: Muenster Epistemic Trustworthiness Inventory.

^d^ROS: recipient orientation scale.

## Discussion

### Principal Findings

The designated audience and technicality of medical jargon, in general, impacted peoples’ assessment of trustworthiness. An expert who used a low level of MTJ was perceived to be less competent but of more integrity, more benevolent, and more accommodative in terms of addressing the audience than an expert who used a high level of medical jargon. In addition, experts in the context of a professional forum were judged to be more trustworthy—respectively, to have more integrity and benevolence—than experts in advisory forums. Furthermore, information was judged to be more credible when medical jargon was accommodative rather than nonaccommodative to the designated audience. Participants judged information written with high amounts of MTJ to be more credible in professional forums, whereas they judged the same information to be less credible in advisory forums. At the same time, participants judged information written with low amounts of MTJ to be more credible in advisory forums, whereas they judged the same information to be less credible in professional forums. Hence, our hypothesis that accommodative language use should lead to higher perceived information credibility, greater expert trustworthiness, and greater perceived accommodation by the expert compared with nonaccommodative language use can be confirmed in terms of information credibility.

### Limitations

Regarding the representation of the sample, it is striking that most participants did not use forums very often. Although unexperienced forum users clearly took into account where information was presented and whether the use of medical jargon was appropriate in a given context, it is unclear whether their unfamiliarity with forum use affected participants’ abilities to adequately assess trustworthiness or credibility. However, information literacy influences perceptions of credibility [6], and more experienced Internet users consider media in general to be more credible [65,66]. Thus, these findings suggest that participants who are more experienced in using forums might have used other factors to apply their judgments than those used by the participants in our sample—regardless of whether their judgments would have been adequate. Therefore, further research is needed to illuminate whether participants who use forums more often would find similar effects.

In addition, the significant interaction in terms of credibility produced only a small effect size. However, research on psychological media effects faces several challenges (as do many other research fields) that usually lead to small effect sizes. These challenges include measuring media exposure in a reliable and valid way, while at the same time considering certain conditional variables [67].

Surprisingly, the supposed appropriateness of forum type and language use did not affect the perceived accommodation by experts. Instead, due to our operationalization of accommodation by experts, participants may have judged an expert’s accommodation as it fits not only to the designated forum audience but also to an undefined group of over-hearers and eavesdroppers, including the participants themselves. However, we assume that participants did not judge each expert’s accommodation in terms of accommodating the specific person who inquired, because due to our reformulation of inquiry it is unlikely that participants expected the expert to address a specific user’s post. Even so, participants might have faced challenges in identifying whether the experts intended to address the designated audiences or the entire public, as online forums are not privately closed [20,68].

### Implications

In terms of trustworthiness, our results indicate a few guidelines for experts who are interested in providing information in online health forums. Results show that, generally, providing information in professional forums appears to be more trustworthy. Moreover, using high amounts of MTJ, generally, makes the expert appear to be more competent but also to have less integrity and be less benevolent. Furthermore, experts should aim to use accommodative language and hence use MTJ more appropriately, as taking the audience into account increased people’s perceived credibility of information provided by experts and could promote information seekers’ understanding of information [10,40,50]. However, further research should be done on whether having experts explicitly declare which audience they intend to address—either a specific online audience or the entire public—can help people to more reliably and accurately assess the expert’s trustworthiness and the credibility of their information. Moreover, future research might include aspects of biased information processing, such as in instances where forum users have more well-defined prior attitudes toward the presented information, which users likely did not have in this study [69]. In terms of the relatively high unexplained statistical variance, which resulted in a small effect size for the credibility effect, future research could further consider the influence of individuals’ differences in or underlying mechanisms of information processing [67].

Especially online, people face several challenges in identifying factors that indicate who should be trusted and on which information to rely. Factors that can be used to assess the credibility of information are, for instance, the author’s credentials, their expertise, and if they use comprehensible language [6,7,35]. In addition, people should also use the context of online communication to make these judgments [2,4]. However, to our knowledge, only few studies have investigated whether users are sensitive to context cues when judging the credibility of online information [70]. Future research, therefore, should not only investigate whether people are sensitive to different online contexts but should also consider cues about online context other than the designated audience.

### Conclusions

The results of our study clearly illustrate that users keep track of wording and the context of information when reading online health information. It is interesting to see that not only did both experimental factors impact the assessment, but so did the interaction of the 2 factors. According to methodology, this study illustrates how specific research questions can be addressed by varying central features of online forums in an experimental study. Thus, this study is able to offer more specific insights on trustworthiness and credibility assessment in online settings than research usually provides [6,71]. By focusing on 2 relevant aspects of online forums (ie, the context, namely, designated audience, and the MTJ), this study helps to assess the effect of appropriateness between language use and the context of online communication. A future challenge will be to specify the appropriateness of language use not only in terms of online audience but also in terms of other aspects of online communication contexts.

## Appendix

Multimedia Appendix 1 Framing of designated audience of forum types (translation from German).

Multimedia Appendix 2 Short forum descriptions (translation from German).

Multimedia Appendix 3 High vs low medical technical jargon for each of the 10 nutrition myths and the introductory question for expert’s posts (translation from German).

Multimedia Appendix 4 Screenshot of a survey site showing an expert’s post.

## Abbreviations

CAT: communication accommodation theory
METI: Muenster Epistemic Trustworthiness Inventory
MTJ: medical technical jargon
ROS: recipient orientation scale
SMS: short message service

## References

[1] Sbaffi L, Rowley J (2017). Trust and credibility in web-based health information: a review and agenda for future researcha. J Med Internet Res.

[2] Eysenbach G, Metzger MJ, Flanagin AJ (2008). Credibility of health information digital media: New perspectives implications for youth. Digital media, youth, credibility.

[3] Modave F, Shokar NK, Peñaranda E, Nguyen N (2014). Analysis of the accuracy of weight loss information search engine results on the internet. Am J Public Health.

[4] Eysenbach G, Diepgen TL (1998). Towards quality management of medical information on the internet: evaluation, labelling, and filtering of information. Br Med J.

[5] Metzger MJ, Flanagin AJ (2013). Credibility and trust of information in online environments: the use of cognitive heuristics. J Pragmat.

[6] Choi W, Stvilia B (2015). Web credibility assessment: conceptualization, operationalization, variability, and models. J Assn Inf Sci Tec.

[7] Sillence E, Briggs P, Harris PR, Fishwick L (2007). How do patients evaluate and make use of online health information?. Soc Sci Med.

[8] Pollard CM, Pulker CE, Meng X, Kerr DA, Scott JA (2015). Who uses the Internet as a source of nutrition and dietary information? An Australian population perspective. J Med Internet Res.

[9] Fox S (2011). Pew Internet.

[10] Bromme R, Jucks R, Schober M, Rapp DN, Britt MA (2017). Discourse and expertise: The challenge of mutual understanding between experts and laypeople. The Routledge Handbook of Discourse Processes.

[11] Mayer RC, Davis JH, Schoorman FD (1995). An integrative model of organizational trust. Acad Manage Rev.

[12] Friedman B, Khan PH, Howe DC (2000). Trust online. Commun ACM.

[13] Kelton K, Fleischmann K, Wallace W (2008). Trust in digital information. J Am Soc Inf Sci.

[14] Hendriks F, Kienhues D, Bromme R (2015). Measuring laypeople's trust in experts in a digital age: the muenster epistemic trustworthiness inventory (METI). PLoS One.

[15] Freeman KS, Spyridakis JH (2004). An examination of factors that affect the credibility of online health information. Tech Commun.

[16] Chiu Y (2011). Probing, impelling, but not offending doctors: the role of the internet as an information source for patients' interactions with doctors. Qual Health Res.

[17] Eysenbach G (2003). The impact of the Internet on cancer outcomes. CA Cancer J Clin.

[18] Hajli MN, Sims J, Featherman M, Love PE (2014). Credibility of information in online communities. JSM.

[19] Kim S, Yoon J (2012). The use of an online forum for health information by married Korean women in the United States. Inf Res.

[20] Anesa P, Fage-Butler A (2015). Popularizing biomedical information on an online health forum. Iberica.

[21] Vennik FD, Adams SA, Faber MJ, Putters K (2014). Expert and experiential knowledge in the same place: patients' experiences with online communities connecting patients and health professionals. Patient Educ Couns.

[22] Johnsen JK, Rosenvinge JH, Gammon D (2002). Online group interaction and mental health: an analysis of three online discussion forums. Scand J Psychol.

[23] Reddit.

[24] Doctors.net.uk.

[25] MedHelp.

[26] Doctors.net.uk.

[27] Inspire.

[28] Dailystrength.

[29] MacLean D, Gupta S, Lembke A, Manning C, Heer J (2015). An analysis of an online Health forum dedicated to addiction recovery.

[30] Nonnecke B, Preece J (2001). Why lurkers lurk. http://aisel.aisnet.org/amcis2001/294.

[31] Jucks R, Linnemann G, Thon F, Zimmermann M, Blöbaum B (2016). Trust the words. Insights into the role of language in trust building in a digitalized world. Trust and Communication in a Digitized World.

[32] Liao W, Bazarova NN, Yuan YC (2016). Expertise judgment and communication accommodation in linguistic styles in computer-mediated and face-to-face groups. Communic Res.

[33] Bromme R, Jucks R, Runde A (2005). Barriers and Biases in Computer-Mediated Knowledge Communication.

[34] Jucks R, Paus E (2011). What makes a word difficult? Insights into the mental representation of technical terms. Metacogn Learn.

[35] Thon FM, Jucks R (2017). Believing in expertise: How authors' credentials and language use influence the credibility of online health information. Health Commun.

[36] Giles H (2016). Communication accommodation theory: Negotiating personal and social identities in context.

[37] Coupland J, Coupland N, Giles H, Henwood K (2008). Accommodating the elderly: invoking and extending a theory. Lang Soc.

[38] Bell A (2008). Language style as audience design. Lang Soc.

[39] Bientzle M, Griewatz J, Kimmerle J, Küppers J, Cress U, Lammerding-Koeppel M (2015). Impact of scientific versus emotional wording of patient questions on doctor-patient communication in an internet forum: a randomized controlled experiment with medical students. J Med Internet Res.

[40] Gasiorek J, Giles H, Soliz J (2015). Accommodating new vistas. Lang Commun.

[41] Giles H, Ogay T, Whaley BB, Samter W (2007). Communication Accommodation Theory. Explaining Communication: Contemporary Theories and Exemplars.

[42] Stevenson FA, Cox K, Britten N, Dundar Y (2004). A systematic review of the research on communication between patients and health care professionals about medicines: the consequences for concordance. Health Expect.

[43] Bientzle M, Fissler T, Cress U, Kimmerle J (2017). The impact of physicians' communication styles on evaluation of physicians and information processing: a randomized study with simulated video consultations on contraception with an intrauterine device. Health Expect.

[44] Kripalani S, Jacobson TA, Mugalla IC, Cawthon CR, Niesner KJ, Vaccarino V (2010). Health literacy and the quality of physician-patient communication during hospitalization. J Hosp Med.

[45] Sim D, Yuan S, Yun J (2016). Health literacy and physician-patient communication: a review of the literature. Int J Commun Health.

[46] Koch-Weser S, Rudd RE, Dejong W (2010). Quantifying word use to study health literacy in doctor-patient communication. J Health Commun.

[47] Saha S, Beach MC (2011). The impact of patient-centered communication on patients' decision making and evaluations of physicians: a randomized study using video vignettes. Patient Educ Couns.

[48] Pollard CM, Howat PA, Pratt IS, Boushey CJ, Delp EJ, Kerr DA (2016). Preferred tone of nutrition text messages for young adults: focus group testing. JMIR Mhealth Uhealth.

[49] Swenson SL, Buell S, Zettler P, White M, Ruston DC, Lo B (2004). Patient-centered communication: do patients really prefer it?. J Gen Intern Med.

[50] Jucks R, Paus E, Bromme R (2012). Patients' medical knowledge and health counseling: what kind of information helps to make communication patient-centered?. Patient Educ Couns.

[51] Bromme R, Jucks R, Wagner T (2005). How to refer to ‘diabetes’? Language in online health advice. Appl Cognit Psychol.

[52] Kimmerle J, Bientzle M, Cress U (2017). “Scientific evidence is very important for me”: the impact of behavioral intention and the wording of user inquiries on replies and recommendations in a health-related online forum. Comput Human Behav.

[53] Jucks R, Bromme R (2007). Choice of words in doctor-patient communication: an analysis of health-related internet sites. Health Commun.

[54] Brown BL, Giles H, Thakerar JN (1985). Speaker evaluations as a function of speech rate, accent and context. Lang Commun.

[55] Soliz J, Giles H (2016). Relational and identity processes in communication: a contextual and meta-analytical review of Communication Accommodation Theory. Annals of the International Communication Association.

[56] Matthes J, Kohring M (2003). Operationalization of trust in journalism [in German]. M&K.

[57] Yang Q, Beatty M (2016). A meta-analytic review of health information credibility: belief in physicians or belief in peers?. HIM J.

[58] Wangberg S, Andreassen H, Kummervold P, Wynn R, Sørensen T (2009). Use of the internet for health purposes: trends in Norway 2000-2010. Scand J Caring Sci.

[59] Trends.google.

[60] de Groot H, Farhadi J (2015). Ernährungswissenschaft.

[61] Wulff HR (2004). The language of medicine. J R Soc Med.

[62] Langer I, Schulz von Thun F, Tausch R (2011). Sich verständlich ausdrücken.

[63] Reuter P (2005). Springer Taschenwörterbuch Medizin.

[64] Jucks R, Becker B, Bromme R (2008). Lexical entrainment in written discourse? Is expert's word use adapted to the addressee?. Discourse Process.

[65] Flanagin AJ, Metzger MJ (2016). Perceptions of internet information credibility. Journal Mass Commun Q.

[66] Zulman DM, Kirch M, Zheng K, An LC (2011). Trust in the internet as a health resource among older adults: analysis of data from a nationally representative survey. J Med Internet Res.

[67] Valkenburg P, Peter J (2013). Five challenges for the future of media-effects research. ‎Int J Commun.

[68] Bernstein M, Bashy E, Burke M, Karrer B (2013). Quantifying the invisible audience in social networks.

[69] Nauroth P, Gollwitzer M, Bender J, Rothmund T (2015). Social identity threat motivates science-discrediting online comments. PLoS One.

[70] Hu Y, Shyam Sundar S (2009). Effects of online health sources on credibility and behavioral intentions. Communic Res.

[71] Sundar S, Metzger MJ, Flanagin AJ (2008). The MAIN Model: A heuristic approach to understanding technology effects on credibility. Digital Media, Youth, and Credibility.

