# Supplementary material for: How Experts’ Use of Medical Technical Jargon in Different Types of Online Health Forums Affects Perceived Information Credibility: Randomized Experiment With Laypersons
Source: J Med Internet Res. 2018 Jan 23;20(1):e30. doi: 10.2196/jmir.8346 (PMC5801514; doi:10.2196/jmir.8346)
Supplement: Multimedia Appendix 1 [file jmir_v20i1e30_app1.pdf]

Appendix A

***Table 1**. Framing of Designated Audience of Forum Types (Translation from German).*

Overall forum introduction to **professional forums** / **advisory forums**[a]
Information about the selected internet forums
The following contributions represent contributions from forums about the topics of health and nutrition. We have always chosen forums, which are mainly used by **medical professionals / patients and other non-experts in these fields**. In these forums, especially **physicians** / **laypeople** use the possibilities to **exchange information on technical content** / **inform themselves about questions** and value **in discussion** is set on information being **scientific** / is set on information **being clearly explained**. In these forums **medical professionals** / **laypeople and non-experts in these fields** are the ones mainly taking part in the discussions. The contributions of these forums can be read by all Internet users. **However,** laypeople and non-specialists **rather write own contributions.** / Laypeople and non-specialists **write most of the contributions on their own and receive answers from professionals**. All answers presented here are given by professionals of these forums.

[a] Differences between professional and advisory forum introductions are highlighted in bold and color.
