# Supplementary material for: How Experts’ Use of Medical Technical Jargon in Different Types of Online Health Forums Affects Perceived Information Credibility: Randomized Experiment With Laypersons
Source: J Med Internet Res. 2018 Jan 23;20(1):e30. doi: 10.2196/jmir.8346 (PMC5801514; doi:10.2196/jmir.8346)
Supplement: Multimedia Appendix 2 [file jmir_v20i1e30_app2.pdf]

Appendix B

Table 1. *Short forum descriptions (Translation from German).*

	Short forum introduction of **professional forums** / **advisory forums**[a]
1	The post stemmed from a **"Physicians-**forum"/ an **"Ask the doctor-**forum". Mainly **physicians discuss about professional topics / laypeople ask questions about the topic**.
2	This post is from a forum where especially **professionals among each other exchange** / **non-experts want to inform themselves** about medical contents.
3	Here is a post from a forum, which is used for **discussions between / questions from laypeople to** healthcare professionals and where mostly **physicians write / laypeople ask questions**.
4	The post was found in a forum where mainly **physicians give each other thematic suggestions** / **laypeople can inform themselves**.
5	Here is a post from a forum for **technical exchange between healthcare professionals** / **questions from non-experts**.
6	This post was found in a medical forum. Mainly **physicians** / **medical laypeople** are registered there.
7	The post is from a forum for **healthcare professionals** / **non-experts**. Mainly **physicians exchange** / **laypeople inform themselves about** medical content here.
8	This post is from a **physician's-forum** / **healthcare-forum** where mainly **healthcare professionals discuss** / **questions are asked by non-experts**.
9	This post is from a **technical** / **help** forum where mainly **physicians post** / **laypeople participate by asking questions**.
10	Here a post from a forum for **professional exchange between physicians** / **medical laypeople's questions**.

[a] Differences between professional and advisory forum introductions are highlighted in bold and color.
