# Supplementary material for: How Experts’ Use of Medical Technical Jargon in Different Types of Online Health Forums Affects Perceived Information Credibility: Randomized Experiment With Laypersons
Source: J Med Internet Res. 2018 Jan 23;20(1):e30. doi: 10.2196/jmir.8346 (PMC5801514; doi:10.2196/jmir.8346)
Supplement: Multimedia Appendix 3 [file jmir_v20i1e30_app3.pdf]

Appendix C

Table 1. *High vs. low medical technical jargon for each of the 10 nutrition myths and the introductory question for expert's posts (Translation from German).*

Introductory Question	high medical technical jargon [a]	low medical technical jargon [a]
1<br><br>To the question of a relationship between coffee consumption and the risk of developing dementia, this expert answered:	Caffeine, contained in coffee, has a **central stimulating** effect, like other **purine bases**, and is said to have many positive and negative **somatic** characteristics.<br>There are **longitudinal** studies **that** identify a 16% reduced risk of being affected by **Alzheimer's disease (varies in German: Morbus Alzheimer)**. However, it is still unclear, if caffeine alone is responsible for it or if other **ingredients** and factors are perhaps also **relevant**. Coffee can therefore only possible **reduce** the risk. In particular, there is not sufficient evidence.	Caffeine, contained in coffee, has a stimulating effect **in the brain**, like other **building blocks of DNA**, and is said to have many positive and negative **physical** characteristics.<br>There are **long-term** studies **to it**. **These** identify a 16% reduced risk of being affected by **Alzheimer's disease (varies in German: Krankheit Alzheimer)**. However, it is still unclear, if caffeine alone is responsible for it or if other **components** and factors are perhaps also **important**. Coffee can therefore only possible **lower** the risk. In particular, there is not sufficient evidence.
2<br><br>To the question of an effect of low salt diet, this expert answered:	There is some evidence that low-sodium **nutrition** works especially **antihypertensive**, **wherefore** it shall be assumed that **arterial hypertension** can lead to **angiocardiopathy** in the long run. The WHO recommends to lower the daily intake of **sodium chloride** by 6 gram. Therefore, the **systolic** blood pressure is supposed to lower from five to six milliliters and the **diastolic one** from one to three milliliters. This effect would then be comparable to a **weight reduction**.	There is some evidence that low-sodium **diet lowers blood pressure**. **Therefore,** it shall be assumed that **high blood pressure** can lead to **heart-diseases and diseased blood vessels** in the long run. The WHO recommends to lower the daily intake of **table salt** by 6 gram. Therefore, the **high** blood pressure is supposed to lower from five to six milliliters and the **low one** from one to three milliliters. This effect would then be comparable to a **weight loss**.
3<br><br>To the question, if drinking light-lemonade harms, this expert answered:	It is known that persons who often drink beverages with **aspartame or cyclamate** are more likely to get **diabetes mellitus** over a long period of time than those who rarely or never drink such drinks, but it cannot be said certainly if these **ingredients** are responsible for it.<br>Possibly humans **prefer** light drinks if they have a **disposition** to **adipositas.**	It is known that persons who often drink beverages with **sweeteners** are more likely to get **diabetes** over a long period of time than those who rarely or never drink such drinks. But it cannot be said certainly if these **additives** are responsible for it.<br>Possibly humans **favour** light drinks if they have a **tendency** to **overweight**.

Introductory Question	high medical technical jargon [a]	low medical technical jargon [a]
4<br><br>To the question, of an effect of vitamin products, this expert answered:	Vitamins are indeed organic components **that** are not **synthesize** sufficiently by humans and need to be taken in with the food in **micro-quantities**. **Calciferol** is the only exception, it is **endogenously synthesized**. In industrial nations a sufficient supply for healthy persons is usually ensured through the **extensive** food offers. **Hypervitaminosis** can instead, depending on the vitamin, lead to different negative effects. And **chronical intoxication** could be caused by **supplementation**.	Vitamins are indeed organic components. **These** are not **produced** sufficiently by humans and need to be taken in with the food in **micro-quantities**. **Vitamin D** is the only exception, it is **produced in the body**. In industrial nations a sufficient supply for healthy persons is usually ensured through **extra rich** food offers. **Overhang of vitamins** can instead, depending on the vitamin, lead to different negative effects. And **long poisoning** could be caused by **addition**.
5<br><br>To the question of harmfulness of eggs, this expert answered:	Likely, this only applies to few people. The yolk is not only quite **lipid-containing**, **it** also contains much cholesterol in particular. A medium-sized egg of 60 g weight provides already 270 mg of the fat-accompanying substance. A **correlation** between the risk for **cardiovascular diseases (**varies in German: **kardiovaskuläre Erkrankungen)** and the consumption of eggs, e.g. in terms of **lipaemia**, could not be determined in any big **observational study (**varies in German: **Observationsstudie)**. Whereas there are indicates for diabetics to such a **correlation** between the consumption of eggs and **cardiovascular diseases (**varies in German: **kardiovaskuläre Erkrankungen).**	Likely, this only applies to few people. The yolk is not only quite **high in fat**. **It** also contains much cholesterol in particular. A medium-sized egg of 60 g weight provides already 270 mg of the fat-accompanying substance. A **relation** between the risk for **cardiovascular diseases (**varies in German: **Herz-Kreislauf-Erkrankungen)** and the consumption of eggs, e.g. in terms of **high fat content in the blood**, could not be determined in any big **observational study (**varies in German: **Beobachtungsstudie)** Whereas there are indicates for diabetics to such a **connection** between the consumption of eggs and **cardiovascular diseases (**varies in German: **Herz-Kreislauf-Erkrankungen).**
6<br><br>To the question of a connection between olive oil and heart diseases, this expert answered:	The regular **ingestion** of extra virgin olive oils can possibly prevent **myocardial infarctions**. Extra virgin olive oil contains higher proportions of desired **ingredients** like **tocopherols**, **carotenoids** and **phytosterols,** but also higher rates of environmental pollutants like **Sulphur (**varies in German: **Sulfur)** and **pesticides**. The **ingredients** tyrosol and **3,4-dihydroxycinnamic acid** showed in **in vitro** investigations reduced secretion of **cytokines (**in German: **Zytokinen**)**, however,** direct proof to **myocardial infarctions** cannot be provided yet.	The regular **consumption** of extra virgin olive oils can possibly prevent **heart attacks.** Extra virgin olive oil contains higher proportions of desired **components** like **vitamin E, precursors of vitamin A** and **cholesterol-lowering sterols,** but also higher rates of environmental pollutants like **Sulphur (**varies in German: **Schwefel)** and **chemicals for pest control**. The **components** tyrosol and **caffeic acid** showed in **test tube** investigations reduced secretion **of egg white which regulates inflammation (**in German: **entzündungsregulierende Eiweiße**). **However,** direct proof to **heart attacks** cannot be provided yet.

Introductory Question	high medical technical jargon [a]	low medical technical jargon [a]
7<br><br>To the question of what happens to a chewing gum when it reaches the bowel, this expert answered:	The **aspiration** of a chewing gum per day is considered harmless, **because** a chewing gum consists in its basic **substance** of indigestible **isobutylene plastics**. But it is simply eliminated, like everything the **gastrointestinal system (**varies in German: **gastrointestinale System**) cannot use. The chewing gum does not adhere to the body, because the milieu in the gastrointestinal tract is **humid** and the **secretion** works as a lubricant. It was merely described a **agglutination** to a bigger ball when small children took in and **aspirated** bigger amounts of chewing gums. This needed to **ektomized** of the **rectum** from a **gastroenterologist**.	The **swallowing** of a chewing gum per day is considered harmless. **Hence,** a chewing gum consists in its basic **component** of **indigestible plastics**. But it is simply eliminated, like everything the **gastrointestinal system (**varies in German: **Magen-Darm-Trakt**) cannot use. The chewing gum does not adhere to the body, because the milieu in the gastrointestinal tract is **moist** and the **mucus** works as a lubricant. It was merely described a **clumping** to a bigger ball when small children took in and **swallowed** bigger amounts of chewing gums. This needed to be **removed** of the **hindgut** from a **doctor**.
8<br><br>To the question, if cheese closes the stomach, this expert answered:	Indeed, cheese does not support the **digestion (**varies in German: **Digestion)** or block the **pars cardiaca**, but you are filled faster when you eat Gouda or Camembert. Responsible for this are **monocarboxylic acids** and **proteins** that are contained in cheese. Fatty foods are more difficult to **digest (**varies in German: **digerieren)**, therefore it takes longer. As a result, a feeling of fullness is triggered in the **ventromedial hypothalamus**. The **gastrointestinal system** also sends a feeling of hunger to the **lateral hypothalamus** later. Cheese therefore does close the stomach in a figurative sense by triggering the release of **neurotransmitters** for a longer time.	Indeed, cheese does not support the **digestion (**varies in German: **Verdauung)** or block the **stomach entrance,** but you are filled faster when you eat Gouda or Camembert. Responsible for this are **essential fatty acids** and **egg whites** that are contained in cheese. Fatty foods are more difficult to **digest (**varies in German: verdauen**)**, therefore it takes longer. As a result, a feeling of fullness is triggered in the **satiety center in the brain**. The **digestive** also sends a feeling of hunger to the **appestat in the brain** later. Cheese therefore does close the stomach in a figurative sense by triggering the release of **biochemical messengers** for a longer time.
9<br><br>To the question of pretzel sticks and cola, this expert answered:	Because cola consists of considerable parts of **saccharin**, the body is additionally dehydrated **causing** an **acute dehydration** when having a lasting disease. Furthermore, cola contains lots of **methyltherobromine**. Although it awakes you, it stimulates the **kidney (**varies in German: **Renes).** The effect is that the body eliminates more potassium than it actual should. Even the popular **combination** "cola with pretzel sticks" does not change anything. Since pretzel sticks only contain **sodium chloride** and cannot fix **the hypokalemia**.	Because cola consists of considerable parts of **sugar**, the body is additionally dehydrated. **This** causes a **rapid drying** when having a lasting disease. Furthermore, cola contains lots of **caffeine**. Although it awakes you, it stimulates the **kidney (**varies in German: **Niere).** The effect is that the body eliminates more potassium than it actual should. Even the popular **mixture** "cola with pretzel sticks" does not change anything. Since pretzel sticks only contain **table salt** and cannot fix **the potassium deficiency**.

Introductory Question	high medical technical jargon [a]	low medical technical jargon [a]
10<br><br>To the question of a connection between healthy nutrition and the protection of cancer, this expert answered:	A balanced nutrition, **sufficient** movement and a normal weight **reduce** the risk to develop **malignancy and cardiovascular diseases** (**varies in German: kardiovaskuläre Erkrankungen**), **obesity**, **arterial hypertonia**, **diabetes mellitus** (varies in German: **Diabetes Mellitus**), and **malignancies** occur rarer to people who are living on a healthy diet. Investigations show that nutrition plays an essential role in the **prophylaxis** of **malignancy**. Avoiding **obesity** is at least equally important.	A balanced nutrition, **enough** movement and a normal weight **lower** the risk to develop **cancer. And cardiovascular** diseases (**varies in German: Herz-Kreislauf-Erkrankgungen**), **overweight**, **high blood pressure**, **diabetes** (varies in German: **Zuckerkrankheit**), and **cancer** occur rarer to people who are living on a healthy diet. Investigations show that nutrition plays an essential role in the **prevention** of **cancer**. Avoiding **overweight** is at least equally important.

[a] *Differences between high and low medical technical jargon are highlighted in bold.*
