# Supplementary material for: How Experts’ Use of Medical Technical Jargon in Different Types of Online Health Forums Affects Perceived Information Credibility: Randomized Experiment With Laypersons
Source: J Med Internet Res. 2018 Jan 23;20(1):e30. doi: 10.2196/jmir.8346 (PMC5801514; doi:10.2196/jmir.8346)
Supplement: Multimedia Appendix 4 [file jmir_v20i1e30_app4.pdf]

Appendix D

Figure 1. Original screenshot of survey site that includes a short description of forum type, a moderated question to introduce the topic, expert's post about this topic, and an excerpt of dependent measures.

Westfälische Wilhelms-Universität Münster

2%

Der Beitrag stammt aus einem ‚Mediziner-Forum'. Hier diskutieren überwiegend Mediziner über fachliche Themen.

Auf die Frage zum Zusammenhang zwischen Kaffeekonsum und dem Risiko an Demenz zu erkranken antwortete diese Fachperson:

Re: Kaffee und Demenz

by »

Posts:
Joined:

Da der in Kaffee enthaltende Stoff Coffein wie andere Bausteine unserer DNA eine stimulierende Wirkung im Gehirn hat, werden ihm tatsächlich allerlei positive und negative körperliche Eigenschaften zugesprochen. Es gibt Langzeitstudien dazu. Diese identifizieren bei Kaffeetrinkern ein um etwa 16% verringertes Risiko an der Krankheit Alzheimer zu erkranken. Es ist allerdings immer noch unklar, ob allein Coffein dafür verantwortlich ist oder ob eventuell andere Inhaltsstoffe und Faktoren wichtig sind. Kaffee kann das Risiko daher nur möglicherweise senken. Es gibt vor allem aber nicht genügend Belege dafür.

Bitte beurteilen Sie die Antwort der Fachperson.

Geben Sie dafür an, inwiefern Sie den folgenden Aussagen zustimmen.

	trifft gar nicht zu	trifft eher nicht zu	ich weiß nicht	trifft eher zu	trifft voll zu
Wie sehr würden Sie der Antwort zustimmen?	○	○	○	○	○
Ich kenne mich gut mit dem Inhalt der Antwort aus.	○	○	○	○	○
Das ist ein komplexes Thema.	○	○	○	○	○
Ich würde Jemandem diese Informationen weitergeben.	○	○	○	○	○
Ich finde die Antwort interessant.	○	○	○	○	○
Ich würde gerne noch jemand Anderes dazu befragen.	○	○	○	○	○
Die Antwort beinhaltet alle notwendigen Informationen.	○	○	○	○	○
Die Antwort gibt die Informationen so wieder, wie sie wirklich sind.	○	○	○	○	○
Ich kann mich auf die Informationen verlassen.	○	○	○	○	○

Wie beurteilen Sie die Eigentschaften der Fachperson?
Bitte geben Sie ihre Einschätzung zu der Fachperson an.

kompetent	○	○	○	○	○	○	○	inkompetent
gerecht	○	○	○	○	○	○	○	ungerecht

Figure 2. Translated screenshot about the topic 'coffee and dementia' showing an accommodative use of high medical technical language in a professional forum.

The post was found in a forum **where mainly physicians give each other thematic suggestions.**

To the question of an effect of low salt diet, this expert answered:

Re: low salt diet

by »

Posts:
Joined:

There is some evidence that low-sodium **nutrition** works especially **antihypertensive, wherefore** it shall be assumed that **arterial hypertension** can lead to **angiocardiopathy** in the long run. The WHO recommends to reduce the daily intake of **sodium chloride** by 6 gram. Therefore the **systolic** blood pressure is supposed to lower from five to six milliliter and the **diastolic one** from one to three milliliter. This effect would then be comparable to a **weight reduction**.

Please, evaluate the response of the expert.

Therefore enter, to which extent do you agree to the following statements.

	strongly disagree	rather disagree	I do not know	rather agree	strongly agree
How much would you agree to the answer?	○	○	○	○	○
The answer contains all necessary information.	○	○	○	○	○
I find the answer interesting.	○	○	○	○	○

Figure 3. Translated screenshot about the topic ‘coffee and dementia’ showing an nonaccommodative use of low medical technical language in a professional forum.

The post was found in a forum **where mainly physicians give each other thematic suggestions.**

To the question of an effect of low salt diet, this expert answered:

Re: low salt diet

by

Posts:
Joined:

There is some evidence that low-sodium **diet reduces blood pressure**. **Therefore** it shall be assumed that **high blood pressure** can lead to **heart-diseases and diseased blood vessels** in the long run. The WHO recommends to reduce the daily intake of **table salt** by 6 gram. Therefore the **high** blood pressure is supposed to lower from five to six milliliter and the **low one** from one to three milliliter. This effect would then be comparable to a **weight loss**.

Please, evaluate the response of the expert.

Therefore enter, to which extent do you agree to the following statements.

	strongly disagree	rather disagree	I do not know	rather agree	strongly agree
How much would you agree to the answer?	○	○	○	○	○
The answer contains all necessary information.	○	○	○	○	○
I find the answer interesting.	○	○	○	○	○
